# Radial Head Fractures

**DOI:** 10.2174/1874325001711011405

**Published:** 2017-11-30

**Authors:** Robert W. Jordan, Alistair DR. Jones

**Affiliations:** 1 University Hospitals Coventry & Warwickshire, UK; 2 Worcester Royal Hospital, Worcester, UK

**Keywords:** Radial head fracture, Radial head replacement, Internal fixation, Elbow, Elbow biomech

## Abstract

**Background:**

Radial head fractures are common elbow injuries in adults and are frequently associated with additional soft tissue and bone injuries.

**Methods:**

A literature search was performed and the authors’ personal experiences are reported.

**Results:**

Mason type I fractures are treated non-operatively with splinting and early mobilisation. The management of Mason type II injuries is less clear with evidence supporting both non-operative treatment and internal fixation. The degree of intra-articular displacement and angulation acceptable for non-operative management has yet to be conclusively defined. Similarly the treatment of type III and IV fractures remain controversial. Traditional radial head excision is associated with valgus instability and should be considered only for patients with low functional demands. Comparative studies have shown improved results from internal fixation over excision. Internal fixation should only be attempted when anatomic reduction and initiation of early motion can be achieved. Authors have reported that results from fixation are poorer and complication rates are higher if more than three fragments are present. Radial head arthroplasty aims to reconstruct the native head and is indicated when internal fixation is not feasible and in the presence of complex elbow injuries. Overstuffing of the radiocapitellar joint is a frequent technical fault and has significant adverse effects on elbow biomechanics. Modular design improves the surgeon’s ability to reconstruct the native joint. Two randomised controlled trials have shown improved clinical outcomes and lower complication rate following arthroplasty when compared to internal fixation.

**Conclusion:**

We have presented details regarding the treatment of various types of radial head fractures - further evidence, however, is still required to provide clarity over the role of these different management strategies.

## 1 INTRODUCTION

**Fig. (1):**
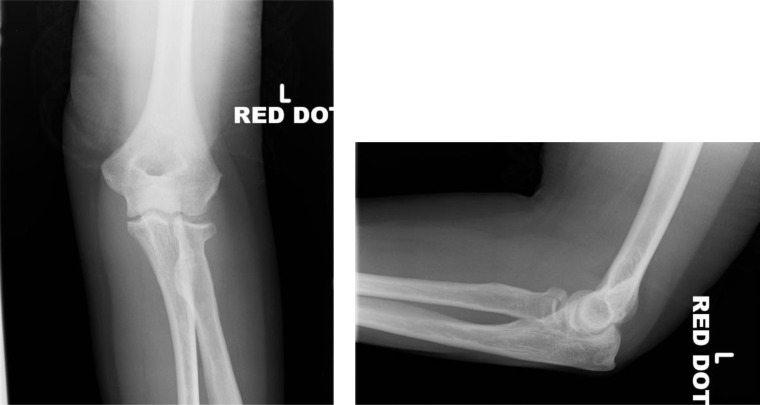
Anteroposterior (AP) and lateral views of a Mason type I injury.

**Fig. (2):**
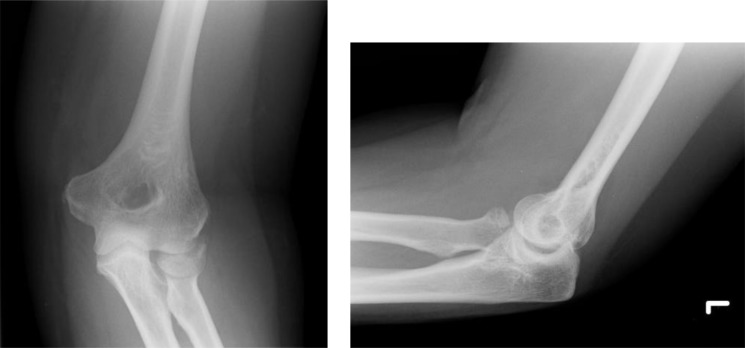
AP and lateral views of a Mason type II injury.

**Fig. (3):**
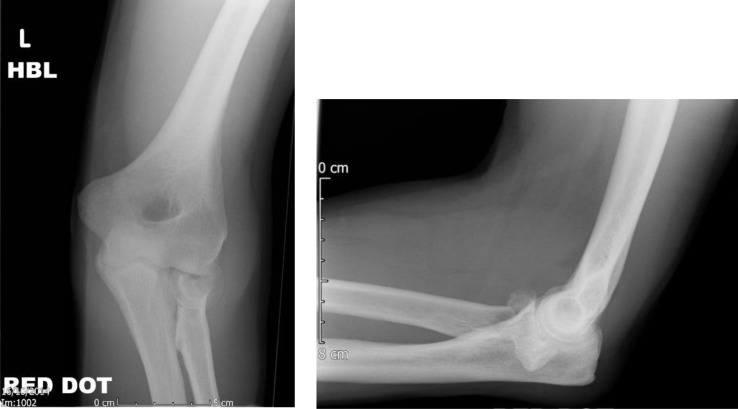
AP and lateral views of a Mason type III injury.

**Fig. (4):**
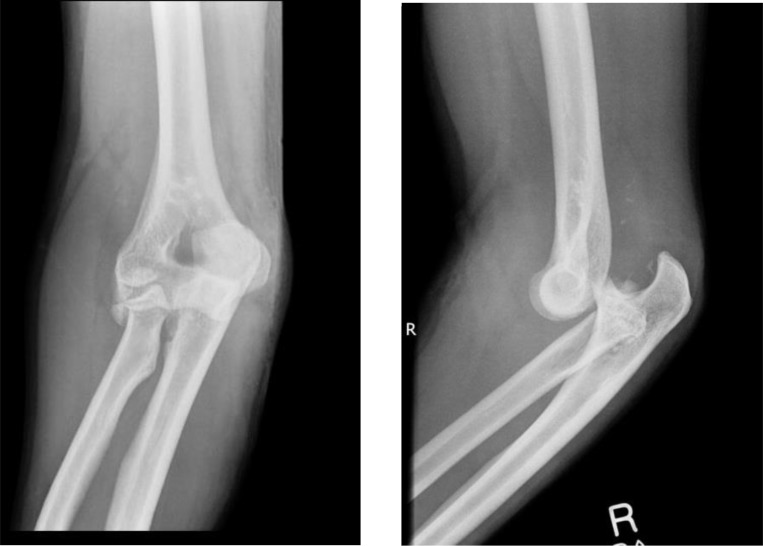
AP and lateral views of a Mason type IV injury.

Radial head fractures constitute 3% of all fractures [1, 2] and 33% of adult elbow fractures [1]. The injury occurs more commonly in females and in middle-aged patients [3]. The mechanism of injury typically involves a fall onto an outstretched hand in a pronated position, which creates an axial load across the elbow. Mason originally classified these fractures [1] and this was later modified by Broberg and Morrey to include the parameters of displacement and size [4]. Table (**1**) illustrates the classification system described by Mason and radiographic examples are demonstrated in (Figs. **1**-**4**).

## 2 ANATOMY

The radial head articulates with both the capitellum and proximal ulna. Anatomical studies have demonstrated that the head is not circular and has variable offset [5]. The radial side of the radiocapitellar joint is convex and covered by articular cartilage. 280 degrees of the rim of the head articulates with the sigmoid notch and is covered by thick hyaline cartilage, whereas the non-articulating arc is covered by thinner cartilage [6, 7]. Successful identification of the non-articular portion allows safe positioning of metalwork as this prevents impingement and restriction of forearm rotation. Various methods have been described to identify this safe zone: marking the middle anteroposterior part of the radial head when in neutral, supination and pronation [8], using the radial styloid and Lister’s tubercle [6] and marking a 110 degree arc from 65 degrees anterolaterally to 45° posterolaterally with the forearm in neutral rotation [6, 8, 9]. The blood supply to the radial head is poor with a single extraosseous vessel entering *via* the bare area responsible in the majority of cases [10]

The radial head is a secondary restraint to valgus force and is responsible for 30% of the support in this direction. Following disruption of the medial collateral ligament (MCL), the radial head is the main stabiliser against valgus and compressive forces [11-13]. Therefore, in the presence of a MCL injury, radial head excision further exacerbates valgus instability. In these cases radial head arthroplasty is capable of restoring stability [14-16]. In addition, the radial head has been shown to act as a constraint to posterolateral [17], axial [18] and varus loading [14]. Restoration of radiocapitellar contact during management is critical to coronal plane and longitudinal stability [19].

## 3 ASSOCIATED INJURIES

Radial head fractures are frequently associated with soft tissue and bone injuries [20]. These injuries will be dependent on mechanism; a valgus compressive force is likely to compromise the medial collateral ligament, whereas a supination injury will injure the lateral collateral followed by anterior and posterior capsule which may include the coronoid process [21]. Radial head fractures that occur in association with ulnohumeral dislocation, coronoid fractures and ligamentous compromise are known as terrible triad injuries. Therefore, it is important to assess for associated injuries both clinically and radiologically. Clinical examination should be inclusive of valgus/varus strain testing and the pivot shift test for posterolateral instability. Computed tomography (CT) can play a role in the detection of concomitant fractures but has only been shown to moderately improve inter-observer reliability in classification of these fractures [22]. Fig. (**5**) demonstrates a reconstructed CT image of a type IV injury and illustrates an associated coronoid fracture. Acute magnetic resonance imaging reveals a high proportion of concomitant injuries; lateral collateral ligament 57%, capitellum injuries 38%, loose osteochondral fragments 5%, medial collateral ligament 2% and coronoid fracture 2% [23]. However, only 5% of these injuries identified on MR were found to be clinically significant during follow up [23].

## 4 NON-OPERATIVE MANAGEMENT

Many fractures of the head of the radius can be treated non-operatively. This includes Mason type I fractures involving less than 25% of the radial head and a small intra-articular step (<2mm). Other authors have reported that even large displaced fractures, which do not interfere with rotation, can be successfully managed nonoperatively with early mobilisation [24, 25]. Retrospective case series with long follow up have reported high levels of satisfaction; Akesson *et al.* reported that 82% of their 49 patients had no subjective complaints at a mean of 19 years; however six patients had required radial head excision for suboptimal outcomes [24]. Duckworth *et al.* noted similar functional outcomes following non-operative and operative fixation of displaced and comminuted radial head fractures [25]. Non-operative treatment typically involves splinting for comfort before early motion to avoid elbow stiffness associated with prolonged immobilisation. One study demonstrated improved functional outcomes after mobilisation at either day one or three compared to mobilisation on day seven [26]. Therefore mobilisation and physiotherapy should be encouraged as early as pain allows when treating radial head fractures non-operatively. Aspiration of the associated intra-articular haematoma reduces pressure and pain and so can be considered as an adjunct to non-operative treatment [27].

## 5 SURGICAL MANAGEMENT

The importance of the radial head to elbow biomechanics and function has led to interest in both its fixation and reconstruction following trauma [19]. The described indications for surgical intervention are variable but include comminution, involvement of a large proportion of articular surface, mechanical block, loose bodies and presence of associated elbow injuries. Previously authors have suggested that the instability of the fracture is probably more important than displacement when considering surgical treatment [28]. However surgical intervention is associated with complications that include posterior interosseous nerve (PIN) injury, loss of fixation, fracture displacement, elbow stiffness, radiocapitellar arthritis and infection. Surgical exposure is typically achieved through either the Kocher interval between anconeus and extensor carpi ulnaris [29] or the Kaplan interval between the extensor carpi radialis longus and extensor digitorum communis [30]. The mean distance from radiocapitellar joint to the PIN ranges from 40 to 48mm and is dependent on arm position. Maintaining the forearm in pronation will move the nerve away from the surgical field and reduce risk of injury [31].

## 6 RADIAL HEAD EXCISION

Radial head excision has been a traditional treatment for comminuted fractures. A number of case series with long-term follow-up have demonstrated that the majority of patients can be treated successfully using this technique. Iftimie *et al.* reported on 51 patients treated for Mason type II to IV fractures followed up for a mean of 17 years and showed that 96% had good or excellent outcome according to the Mayo Elbow Performance Score [32]. A systematic review included four studies and concluded that the majority of patients undergoing radial head excision have a good functional result. However an increase in both valgus angle (range 5 to 11 degrees) and proximal radial migration (range 1.5 to 2.5mm) was noted [33]. Antuna *et al.* reported the outcomes of patients undergoing surgery under the age of 40 years at a mean of 25 years follow up and showed that 92% had good or excellent outcomes, although the authors demonstrated that a high proportion had radiographic evidence of arthritis or some degree of instability on examination [34]. Despite these results concerns regarding the long-term effects of radial head excision have been raised. Excision compromises the stability of the elbow [14, 35], quantified by a cadaveric study as an increased posterolateral laxity of 18.6 degrees [36].

Radial head excision still provides a treatment option for these injuries but is typically reserved for low demand and sedentary patients or after failed alternative management. Contraindications to excision include a coronoid fracture, MCL deficiency and forearm interosseous ligament injury. In addition some authors have recommended avoidance of excision of greater than 25% of radial head because of the risk of painful clicking or symptomatic instability [37, 38].

## 7 OPEN REDUCTION AND INTERNAL FIXATION

Ideally this should only be attempted when anatomic reduction, restoration of congruity and initiation of early motion can be achieved [19]. An example case is illustrated in Figs. (**6** and **7**) where a type IV injury has been managed by reconstruction and fixation of the radial head. This can be easier to achieve with simple fracture patterns, for example in Mason type II fractures where good outcomes have been described [38]. Some studies reporting both type II and III fractures have also shown good or excellent outcomes [39]. However complex fractures are associated with high early failure, non-union and complications [40, 41]. Ring *et al.* concluded that ORIF may be less optimal with greater than three fragments [28]. Comparison of different techniques that included mini-screws, fine threaded wires, Kirschner wires and T-mini-plates demonstrated that threaded wires were optimal for fracture reduction and T-mini-plates provided the least stability [42]. Therefore internal fixation should be considered in radial head fractures with three or fewer fragments but if additional injuries are present these should be addressed separately.

## 8 RADIAL HEAD ARTHROPLASTY

The aim of radial head arthroplasty is reconstruct the anatomy of the native head. The anatomy of the radial head is complex with an elliptical shape, a complex angulation at the radial neck and articulation with the proximal ulna, capitellum and lateral tip of the trochlea [5].

Although various prosthetic materials have been used, the commonest have been silicone and metal. Concerns have been raised regarding silastic implants including fragmentation with resultant synovitis and joint erosion [43]. Silicone implants also provide suboptimal stability against valgus and compressive forces [12, 44-46]. Metal prostheses have been shown to have a higher compressive rigidity and are capable of resisting forces produced across the elbow [12, 46, 47]. Implants are available in monobloc and modular systems. Traditional monobloc designs are technically more demanding [48] whereas modular systems allow the surgeon to alter the height and diameter to facilitate accurate reconstruction [49]. A bipolar design is also available and theoretically improves capitellar tracking, increases radiocapitellar contact areas, and decreases joint contact pressures [16, 50]. Bipolar implants have not been shown to provide superior stability [51] and can be associated with dissociation [52], maltracking [50] and osteolysis [53]. Uncemented prosthesis allows the stem to rotate within the medullary canal effectively creating a bipolar prosthesis [49]. These have also had complications reported, with a study assessing press-fit radial head prostheses concluding that early loosening is common, and often leads to severe osteolysis requiring prosthetic removal [54].

Arthroplasty is indicated for comminuted fractures, especially those with 3 or more fragments where internal fixation is not feasible and in the presence of complex elbow injuries [19]. An example of a type III fracture managed by arthroplasty is illustrated in Figs. (**8** and **9**). A number of case series have demonstrated that this technique can be effective; Grewal *et al.* retrospectively reviewed 26 patients using a modular, metallic, monobloc arthroplasty with a loose press-fit stem and showed high satisfaction, mean DASH 24.4 and mean Mayo Elbow Performance Index (MEPI) of 83, and no revision surgery at two years [55]. Zunkiewicz *et al.* evaluated 29 patients treated using a bipolar prosthesis with a loose stem and a mean follow-up of 34 months, and reported a mean MEPI score of 92 with only two patients requiring further surgery [56].

## 9 SURGICAL PITFALLS IN ARTHROPLASTY

The most common technical fault when performing arthroplasty is overstuffing of the radiocapitellar joint. Shortening or lengthening by as little as 2.5mm can have a significantly adverse effect on elbow biomechanics [57]. A discrepancy can lead to high capitellar contact pressures, capitellar erosion, instability and restricted range of motion [14, 15, 33, 57-60]. To aid sizing, the implant can be checked against the reconstructed native head or by using the proximal edge of the lesser sigmoid notch as a marker for the proximal end of the radial head prosthesis [61, 62].

## 10 COMPLICATIONS

Complications of radial head arthroplasty include radiocapitellar arthritis, stem loosening, failed and/or fractured components, instability, dislocation, and infection [59]. A recent systematic review reported on 12 case series and reported revision rates ranging from 0 to 20%, although the methodology of these studies varied as did the length of follow up [33]. Specific complication rates included peri-prosthetic radiolucency from 0 to 100% and capitellar erosion from 0 to 72% [33]. Duckworth *et al.* recently reported revision and removal rate of 28% at a mean of 6.7 years after uncemented monopolar prosthesis for acute fractures [63]. The authors demonstrated that a lower age and the use of silastic implants were independent risk factors for revision [63]. Similarly van Riet *et al.* evaluated 47 cases of failed metallic radial head prosthesis and reported that common causes for revision were painful aseptic loosening (66%) and instability (19%) [64].

## 11 DISCUSSION

Currently, there is a lack of clear evidence available regarding the treatment of Mason II fractures [49, 65]. Kaas *et al.* reported that non-operative treatment was successful in 80% compared to 93% after ORIF [49] whilst Struijs *et al.* demonstrated residual pain in 42% of non-operative patients versus 32% in surgically treated patients [65]. The Morrey modification added the presence of a mechanical block or greater than 2mm displacement as indications for surgery [66]. However evidence is available that contradicts this with fixation failing to improve outcomes in 2 or 3mm of displacement [67] and a high subjective satisfaction in 82% of patients with 2 to 5mm displaced type II fractures at a mean 19 years follow up [24]. Few comparative studies are published and being non-randomised retrospective designs they have inherent weaknesses. Yoon *et al.* compared 30 cases of partial articular fracture with 2-5mm displacement managed non-operatively with 30 undergoing internal fixation and showed similar function, strength and range of motion [68]. In contrast Zarattini *et al.* reported improved functional scores, residual pain, range of motion and strength after internal fixation when compared to radial excision [69].

The treatment of type III and IV fractures also remains controversial [70]. These patients should be considered to have a complex instability injury due to the high rate of associated injuries, which should be simultaneously addressed for a successful outcome [49]. Traditional radial head excision is associated with valgus instability, stiffness and proximal radial migration [71, 72] and should be considered only for patients with low functional demands. Two non-randomised studies compared excision and internal fixation for comminuted displaced fractures and reported improved functional outcomes and a lower prevalence of arthritis after fixation [73, 74]. Two randomised controlled trials comparing internal fixation and arthroplasty for unstable, multi-fragmented radial head fractures showed a better clinical result and lower complication rate following arthroplasty [75, 76].

## CONCLUSION

The importance of the radial head in elbow biomechanics is becoming increasingly recognised and this has influenced the approach to fracture management. Type I fractures are managed non-operatively. The management of type II and III injuries remains controversial and further evidence in these areas is required. Factors to consider when making management decisions include the ability to achieve a successful reconstruction, elbow stability and the presence of associated injuries.

## Figures and Tables

**Fig. (5):**
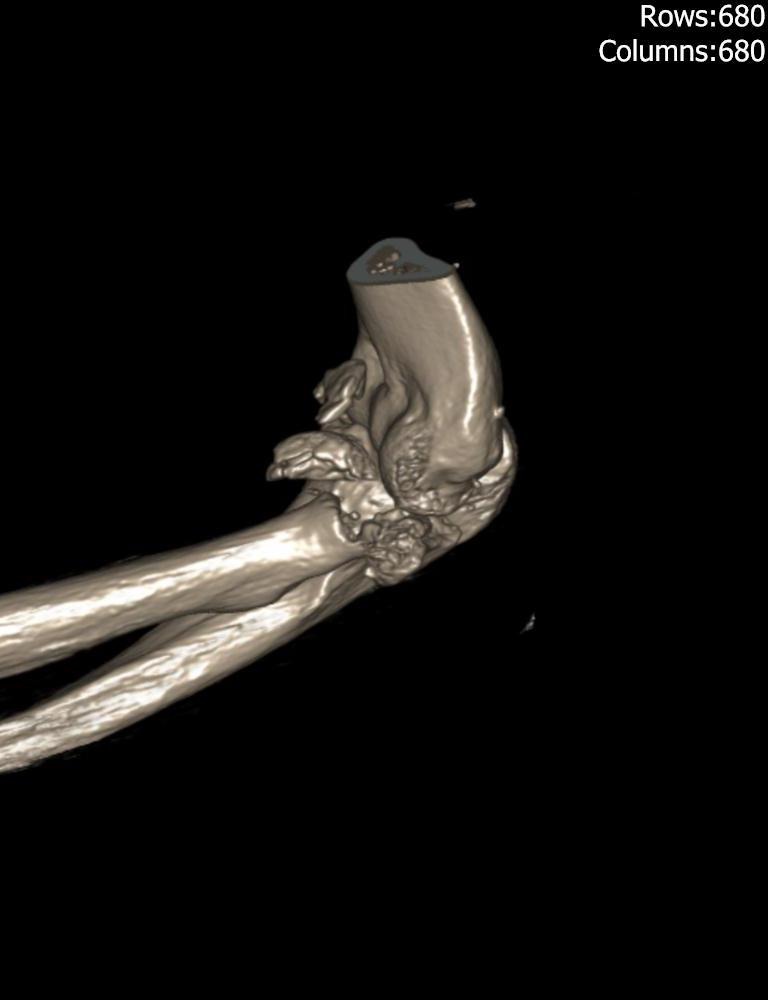
Reconstructed CT image of a type IV injury with an associated coronoid fracture.

**Fig. (6):**
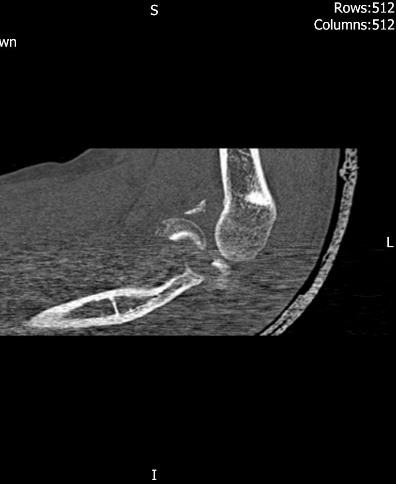
Pre-operative CT images of type IV injury.

**Fig. (7):**
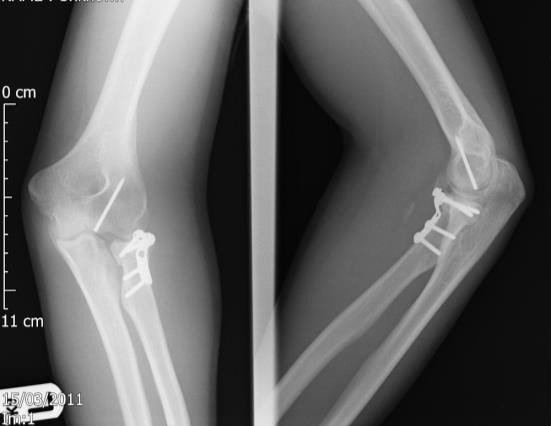
Post-operative plain radiographs after reconstruction and internal fixation of type IV radial head fracture.

**Fig. (8):**
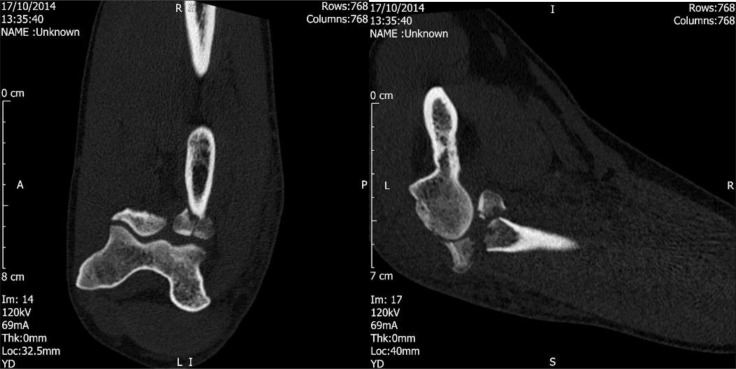
Pre-operative CT images of type III injury.

**Fig. (9):**
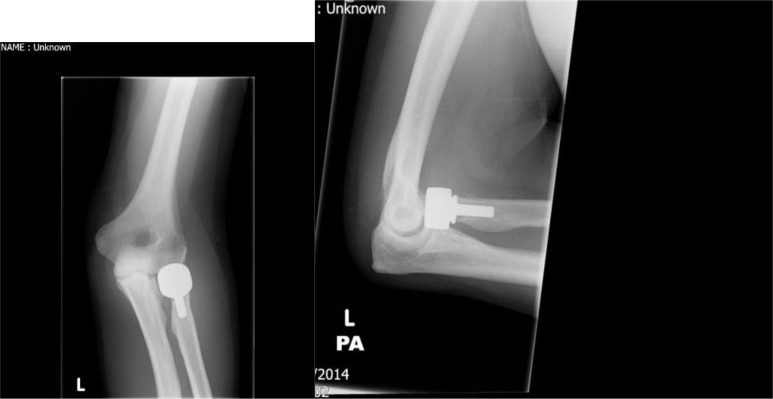
Post-operative plain radiographs after arthroplasty of type III fracture.

**Table 1:** Mason classification of radial head fractures [1].

Mason Classification	Fracture Characteristics
Type I	Undisplaced segmental/marginal fracture Intra-articular displacement <2mm
Type II	Displaced segmental fracture Intra-articular displacement >2mm or angulated
Type III	Comminuted fracture
Type IV	Fracture associated with posterior dislocation
